# Correction to: Comparison of multiple transcriptomes exposes unified and divergent features of quiescent and activated skeletal muscle stem cells

**DOI:** 10.1186/s13395-018-0165-y

**Published:** 2018-06-06

**Authors:** Natalia Pietrosemoli, Sébastien Mella, Siham Yennek, Meryem B. Baghdadi, Hiroshi Sakai, Ramkumar Sambasivan, Francesca Pala, Daniela Di Girolamo, Shahragim Tajbakhsh

**Affiliations:** 1 Bioinformatics and Biostatistics Hub, C3BI, USR 3756 IP CNRS, Institut Pasteur, 75015 Paris, France; 2 Stem Cells and Development, Department of Developmental and Stem Cell Biology, Institut Pasteur, 75015 Paris, France; 3 CNRS UMR 3738, Institut Pasteur, 75015 Paris, France; 4 Institute for Stem Cell Biology and Regenerative Medicine, GKVK PO, Bellary Road, Bengaluru, 560065 India; 5 Dipartimento di Medicina Clinica e Chirurgia, Università degli Studi di Napoli Federico II, Via S. Pansini 5, 80131 Naples, Italy; 6 Novo Nordisk Foundation Center for Stem Cell Biology, DanStem, University of Copenhagen, 3B Blegdamsvej, DK-2200 Copenhagen N, Denmark

## Correction

After publication of this article [1], the authors noted that the legends for Additional file 3: Figure S3 and Additional file 4: Figure S4 were truncated in the production process, therefore lacking some information concerning these Figures. The complete legends are included in this Correction. The authors apologize for any inconvenience that this might have caused.

## Additional files

Additional file 3: **Figure S3.** Effect of adding NICD[E17.5/E14.5] dataset on the best combinations of datasets. Best combination of datasets was determined by the bigger overlap between n (= degree) datasets. n varies from 1 (the dataset containing the most DEGs) to 10 (all the datasets except NICD[E17.5/E14.5]). Blue bars/numbers indicate the number of best overlap between the n datasets, orange bars/numbers indicate, the extent of overlap when NICD[E17.5/E14.5] was added to this best combination. (PDF 395 kb)

Additional file 4: **Figure S4.** Effect of PFA treatment at different time points in the experimental procedure. Top: Schematic showing the simplified experimental procedure of cell fixation before or after muscle dissociation/cell sorting. “0 h” refers to cells fixed prior to muscle dissociation and cell sorting, while “5 h” refers to cells that undergo muscle dissociation and cell sorting prior any treatment (+/− PFA). Bottom: Barplots showing the effect of PFA treatment after muscle dissociation/cell sorting. Bars represent the fold change (in Log10) of expression between freshly isolated QSCs (0 h + PFA) and QSCs after muscle dissociation/cell sorting with or without PFA treatment, 5 h + PFA and 5 h –PFA, respectively. (PDF 445 kb)

## References

[1] Pietrosemoli N, Mella S, Yennek S (2017). Comparison of multiple transcriptomes exposes unified and divergent features of quiescent and activated skeletal muscle stem cells. Skelet Muscle.

