# Supplementary figures and images for: Comparison of multiple transcriptomes exposes unified and divergent features of quiescent and activated skeletal muscle stem cells
Source: Skelet Muscle. 2017 Dec 22;7:28. doi: 10.1186/s13395-017-0144-8 (PMC5741941; doi:10.1186/s13395-017-0144-8)

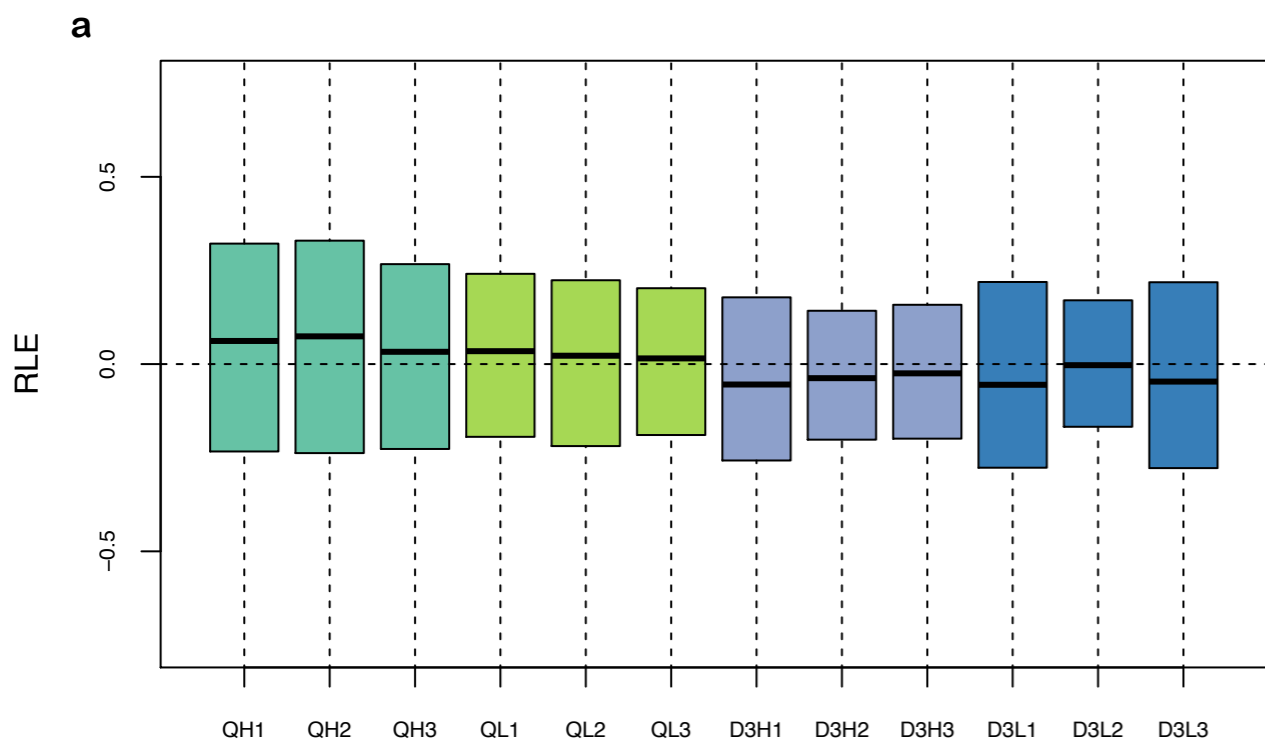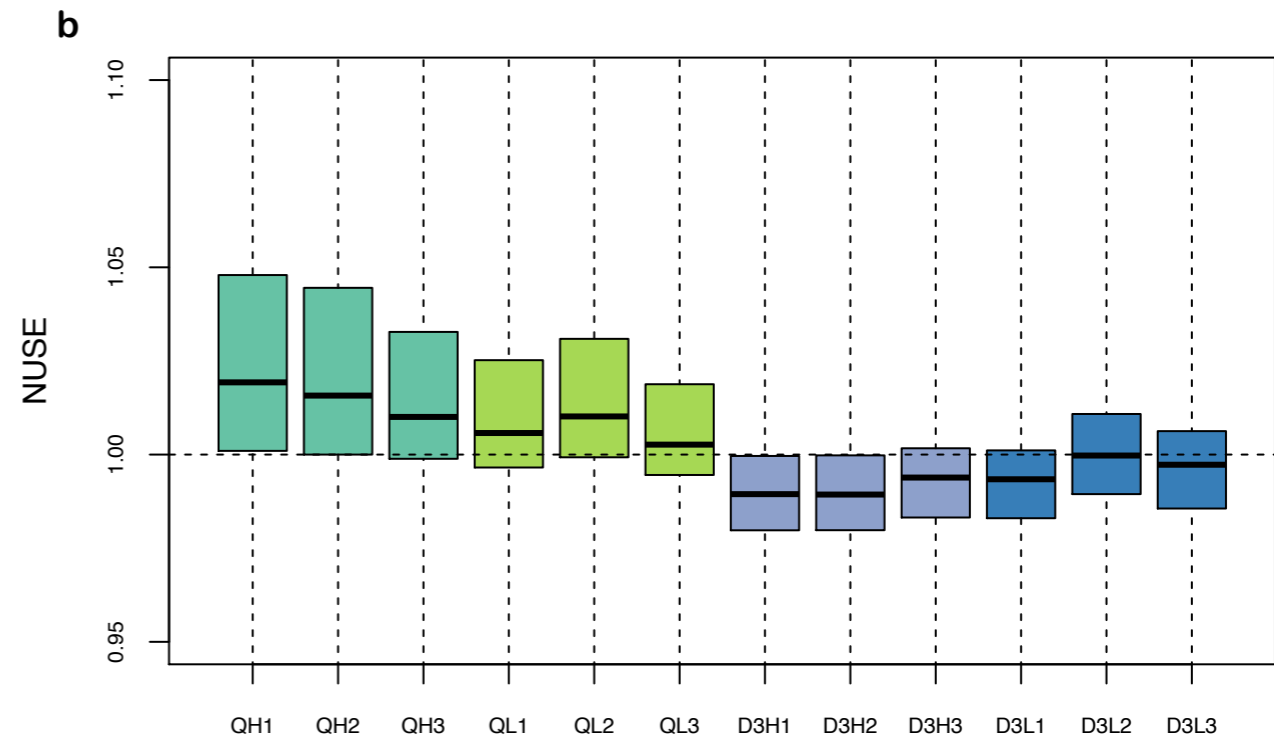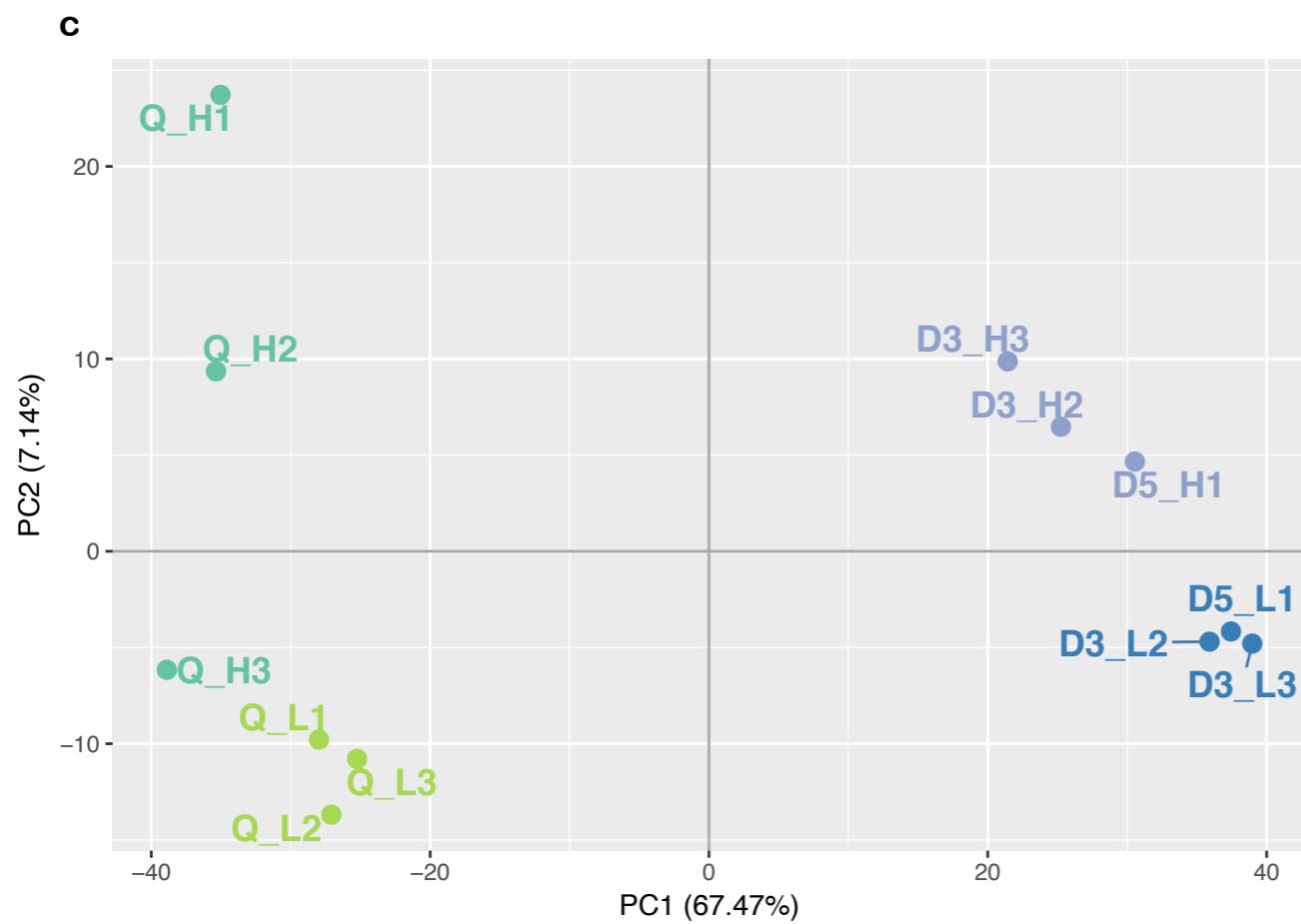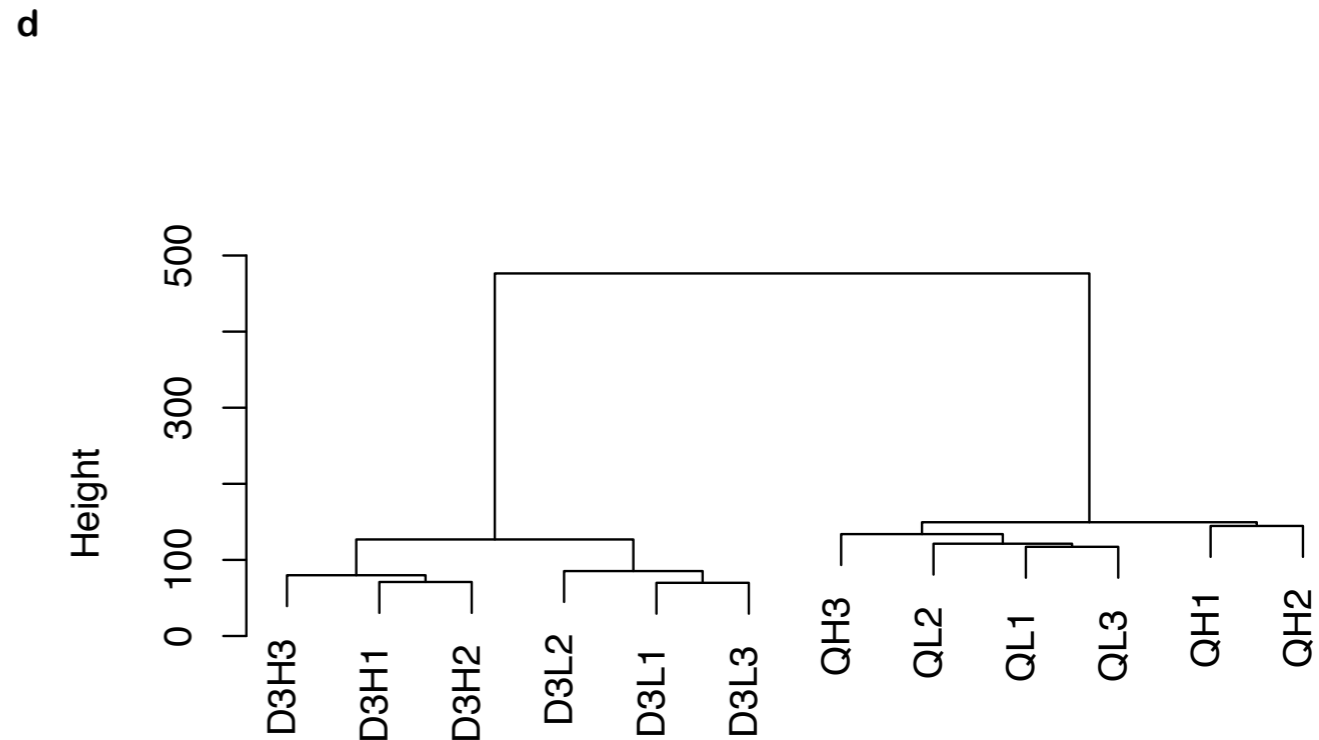

Supplement: Supplementary file 1 — Quality controls and data sample distribution for Quiescent [high/low]/D3Activated [high/low] dataset. a Relative log expression (RLE) and b normalized unscaled standard errors (NUSE) plots for the D3P7 dataset show that as expected for good quality data, RLE median values are centered around 0.0, while the median standard error should be 1 for most genes in the NUSE plots. A sample distribution is distributed according to status (D3H: activated, high; D3L: activated, low; QH: quiescent, high; QL: quiescent, low) using principal component analysis (c) and hierarchical clustering of the Euclidean distance (d). (PDF 103 kb) [file 13395_2017_144_MOESM1_ESM.pdf]

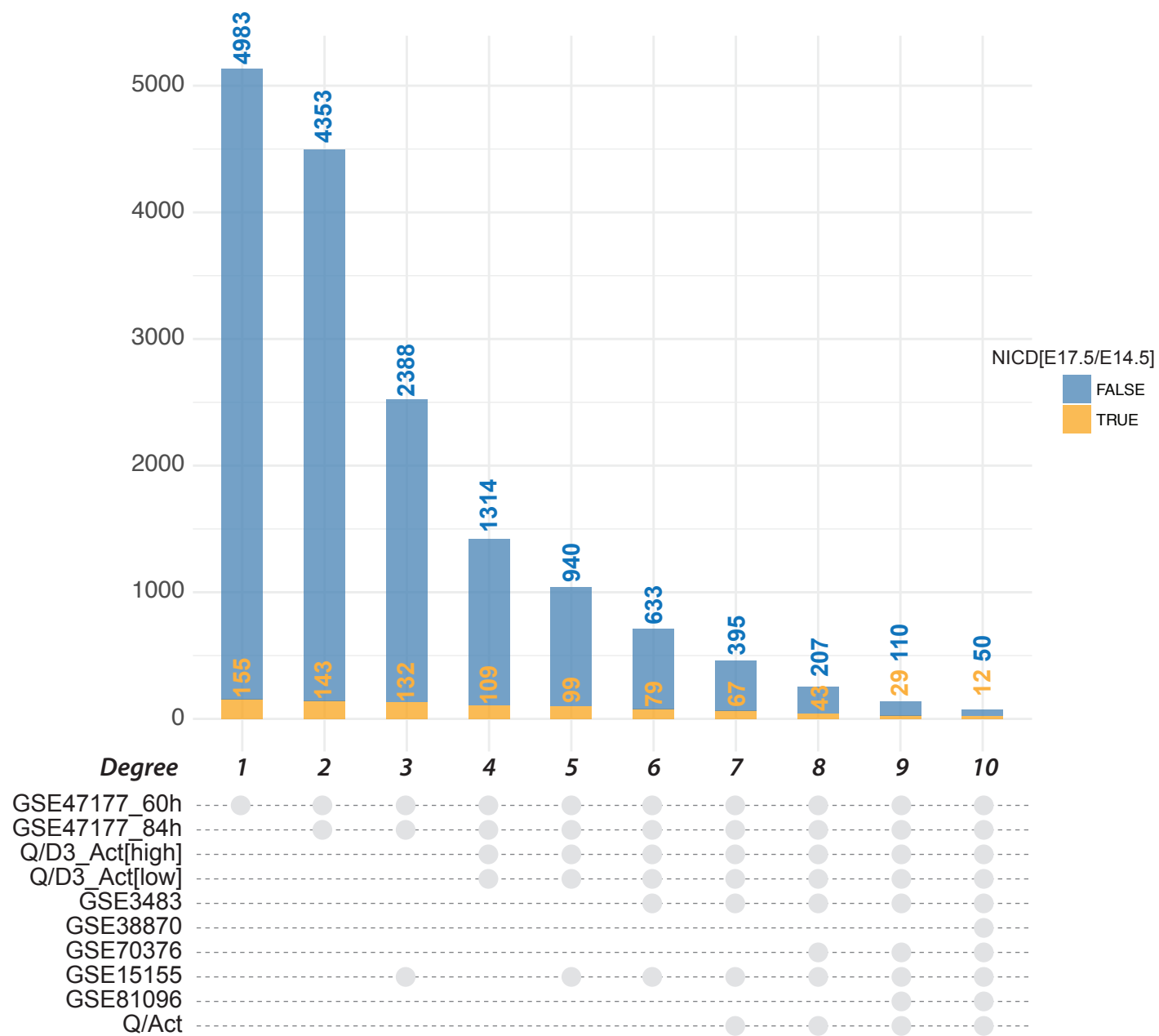

Supplement: Supplementary file 3 — Effect of adding NICD[E17.5/E14.5] dataset on the best combinations of datasets. Impact of including or excluding NICD dataset on overall analysis. (PDF 395 kb) [file 13395_2017_144_MOESM3_ESM.pdf]

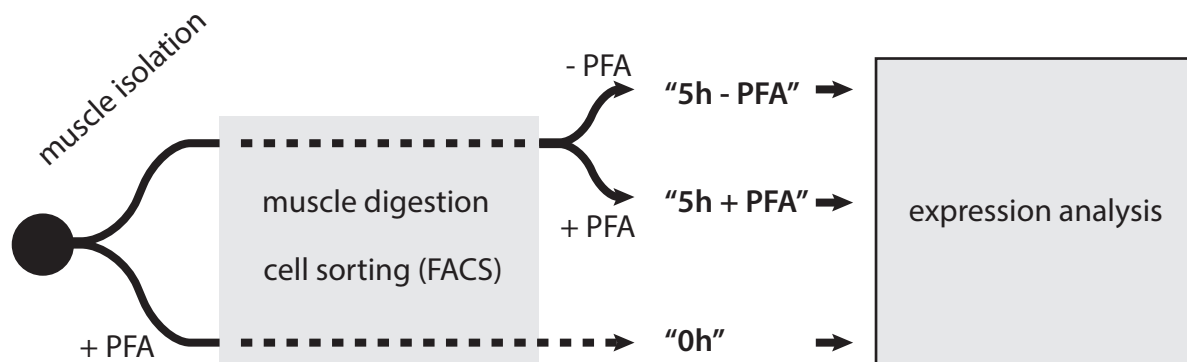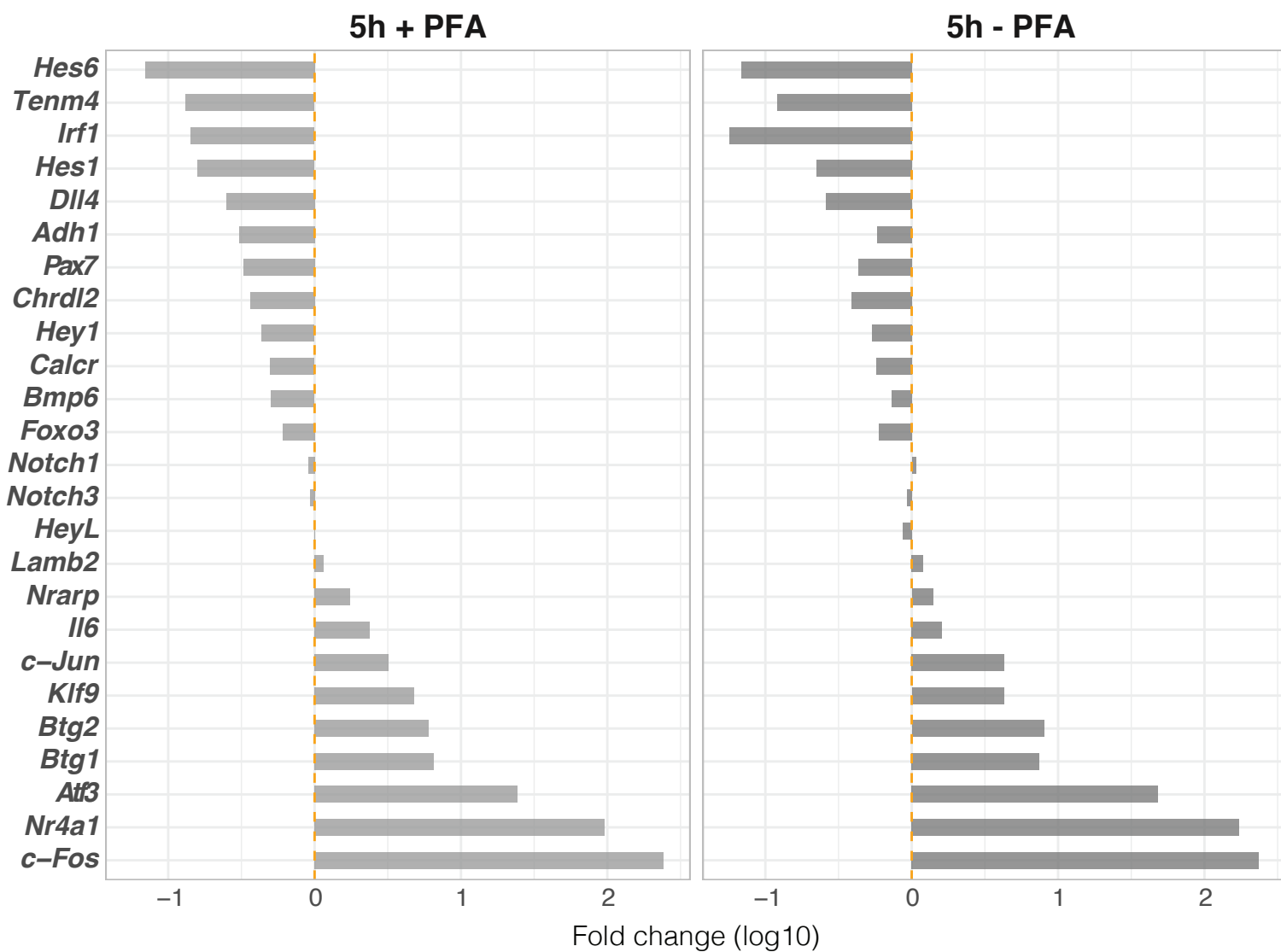

Supplement: Supplementary file 4 — Effect of PFA treatment at different time points in the experimental procedure. Control experiments showing no effect of PFA on gene expression measurements. (PDF 445 kb) [file 13395_2017_144_MOESM4_ESM.pdf]
