# Supplementary material for: Comparison of multiple transcriptomes exposes unified and divergent features of quiescent and activated skeletal muscle stem cells
Source: Skelet Muscle. 2017 Dec 22;7:28. doi: 10.1186/s13395-017-0144-8 (PMC5741941; doi:10.1186/s13395-017-0144-8)
Supplement: Supplementary file 2 — Violin plots of the logFC distribution for each individual dataset. Density plots of the logFC (|logFC| < 1 in red; |logFC| > 1 in blue. (PDF 156 kb) [file 13395_2017_144_MOESM2_ESM.pdf]

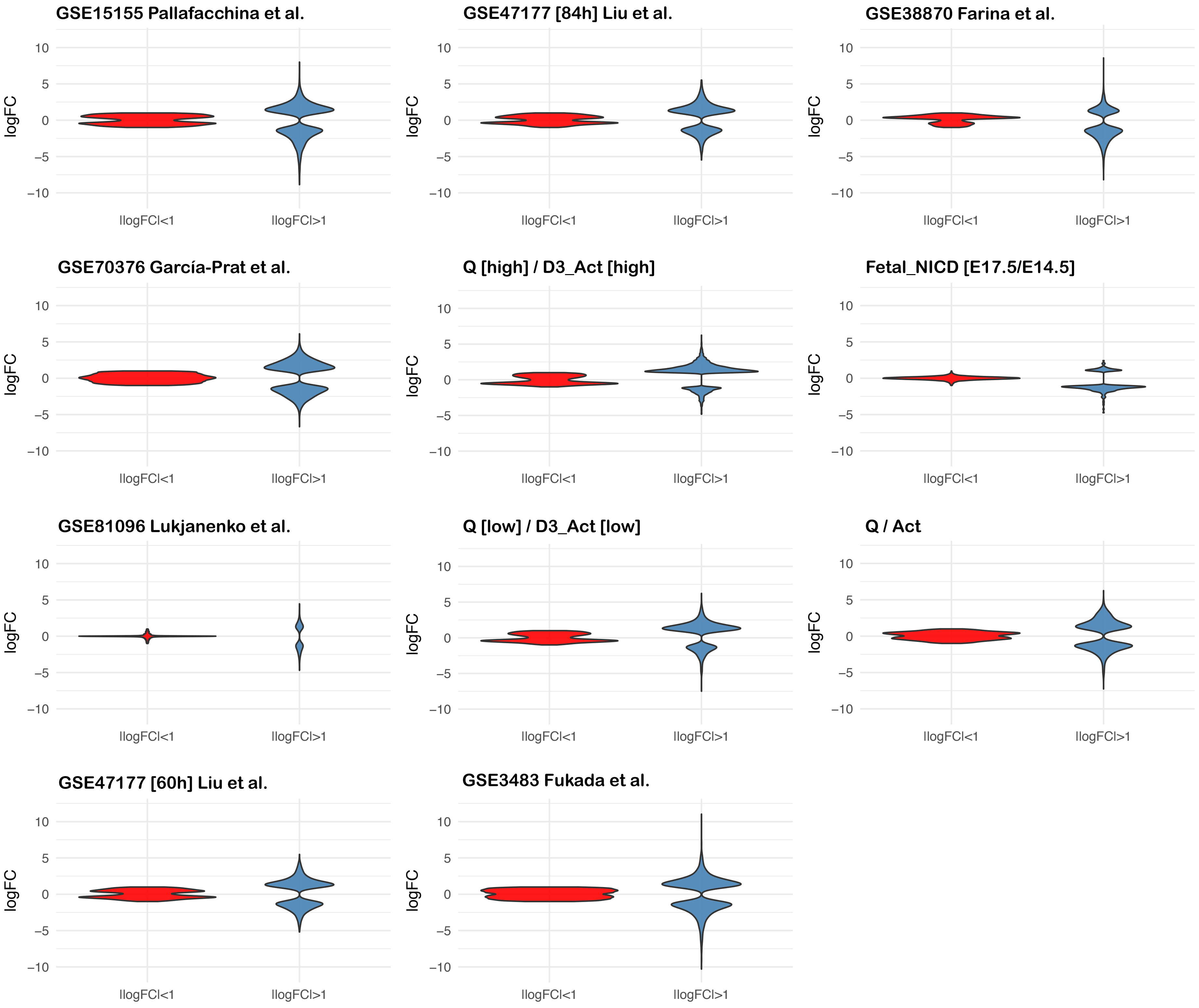

GSE15155 Pallafacchina et al.
GSE47177 [84h] Liu et al.
GSE38870 Farina et al.
GSE70376 García-Prat et al.
Q [high] / D3_Act [high]
Fetal_NICD [E17.5/E14.5]
GSE81096 Lukjanenko et al.
Q [low] / D3_Act [low]
Q / Act
GSE47177 [60h] Liu et al.
GSE3483 Fukada et al.
logFC
10
5
0
−5
−10
|logFC|<1
|logFC|>1
