# Supplementary material for: Comparison of multiple transcriptomes exposes unified and divergent features of quiescent and activated skeletal muscle stem cells
Source: Skelet Muscle. 2017 Dec 22;7:28. doi: 10.1186/s13395-017-0144-8 (PMC5741941; doi:10.1186/s13395-017-0144-8)
Supplement: Supplementary file 6 — Primers used for validation of gene expression by RT-qPCR. Primers used for RT-qPCR studies in Fig. 7. (PDF 14 kb) [file 13395_2017_144_MOESM6_ESM.pdf]

**Gene**	**Forward**	**Reverse**
Adh1	GCAAAGCTGCGGTGCTATG	TCACACAAGTCACCCCTTCTC
Atf3	TTGTTTCGACACTTGGCAGC	TAAACACCTCTGCCATCGGA
Bmp6	TCACCACCCACAGATTGCTA	ACTGTGTGGTGGGGAGTTTT
Btg1	GCGGTGTCCTTCATCTCCAA	GTAACCTGATCCCTTGCACG
Btg2	ACCTTGCTGATGATGGGGTC	GGGTTTCCTCTCCAGTCTCC
Calcr	ATGAGGTGCAAGTCACCCTG	ACTAACTACGCGGTTGGTGG
Chrdl2	GATAGGACTTTTGAGAAGCGG	ACCAGTATCAGTAAGAGCAGC
Dll4	CAGTTGCCCTTCAATTTCACCT	AGCCTTGGATGATGATTTGGC
Foxo3	TCACCCATGCAGACTATCCA	GTCTGGTTGCCGTAGTGTGA
Hes1	ACACCGGACAAACCAAAGAC	AATGCCGGGAGCTATCTTTC
Hes6	CGCACGGATCAACGAGAGT	TCTAGCTTGGCCTGCACCTC
Hey1	CACCTGAAAATGCTGCACAC	ATGCTCAGATAACGGGCAAC
HeyL	GTCTTGCAGATGACCGTGGA	CTCGGGCATCAAAGAACCCT
Il6	GGTCTTGGTCCTTAGCCACT	ACCATAGCTACCTGGAGTACA
Irf1	CCTGAATAGAGGGCGGCAG	CAGCTGCAAAGAGGAACCAG
Klf9	TGGCTGTGGGAAAGTCTATG	AGCGCGAGAACTTTTTAAGG
Lamb2	GAACTTCGCTTGGGCCTACTT	GGTGGCTGGATAGCAGCTT
Notch1	GGTCGCAACTGTGAGAGTGA	TTGCTGGCACATTCATTGAT
Notch3	GTCCAGAGGCCAAGAGACTG	CAGAAGGAGGCCAGCATAAG
Nr4a1	GAGGCTGCTTGGGTTTTGAA	AAAGCGCCAAGTACATCTGC
Nrarp	TGTATCCAGCGTTGTGAAGG	ATTGGGGAAGGCAGAAAGAG
Pax7	GACAAAGGGAACCGTCTGGAT	TGTGAACGTGGTCCGACTG
Rpl13a	GTGGTCCCTGCTGCTCTCAAG	CGATAGTGCATCTTGGCCTTTT
Tenm4	GAGAAATCGATGTGGGGAGA	TAGGAGCCTTCTTCCATCCA
c-Fos	CGGGTTTCAACGCCGACTA	TTGGCACTAGAGACGGACAGA
c-Jun	CCTTCTACGACGATGCCCTC	GGTTCAAGGTCATGCTCTGTTT
JunB	TCACGACGACTCTTACGCAG	CCTTGAGACCCCGATAGGGA
JunD	GAAACGCCCTTCTATGGCGA	CAGCGCGTCTTTCTTCAGC
