# Supplementary figures and images for: Correction to: Comparison of multiple transcriptomes exposes unified and divergent features of quiescent and activated skeletal muscle stem cells
Source: Skelet Muscle. 2018 Jun 6;8:19. doi: 10.1186/s13395-018-0165-y (PMC5991463; doi:10.1186/s13395-018-0165-y)

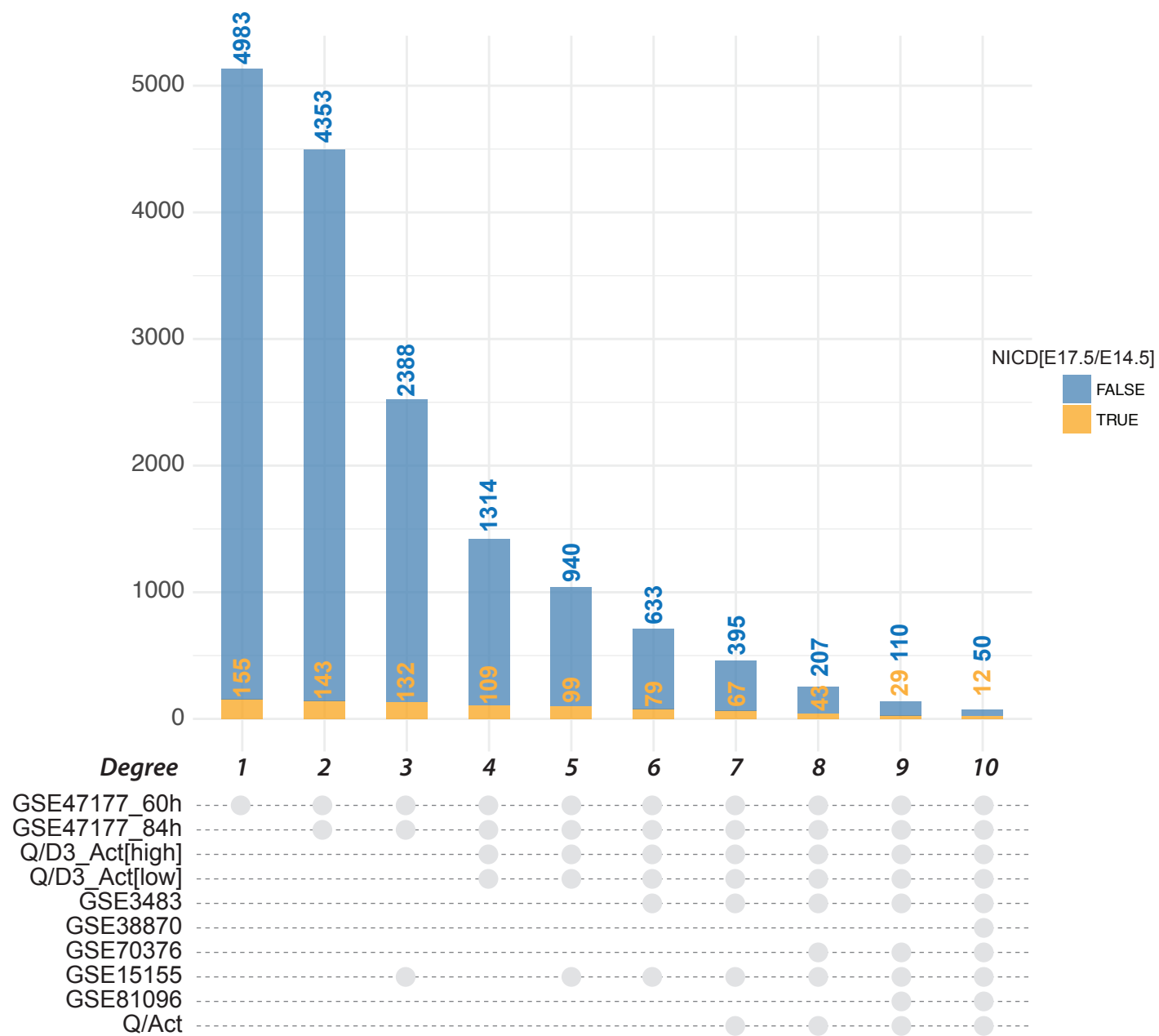

Supplement: Supplementary file 1 — Figure S3. Effect of adding NICD[E17.5/E14.5] dataset on the best combinations of datasets. Best combination of datasets was determined by the bigger overlap between n (= degree) datasets. n varies from 1 (the dataset containing the most DEGs) to 10 (all the datasets except NICD[E17.5/E14.5]). Blue bars/numbers indicate the number of best overlap between the n datasets, orange bars/numbers indicate, the extent of overlap when NICD[E17.5/E14.5] was added to this best combination. (PDF 395 kb) [file 13395_2018_165_MOESM3_ESM.pdf]

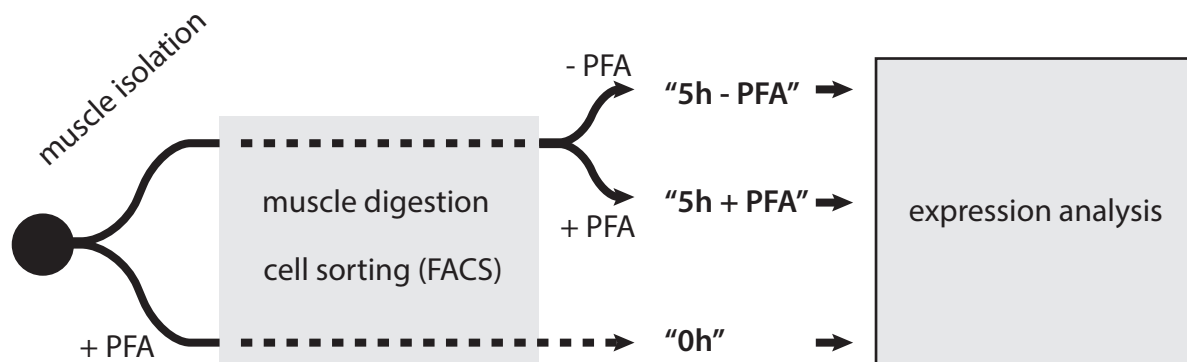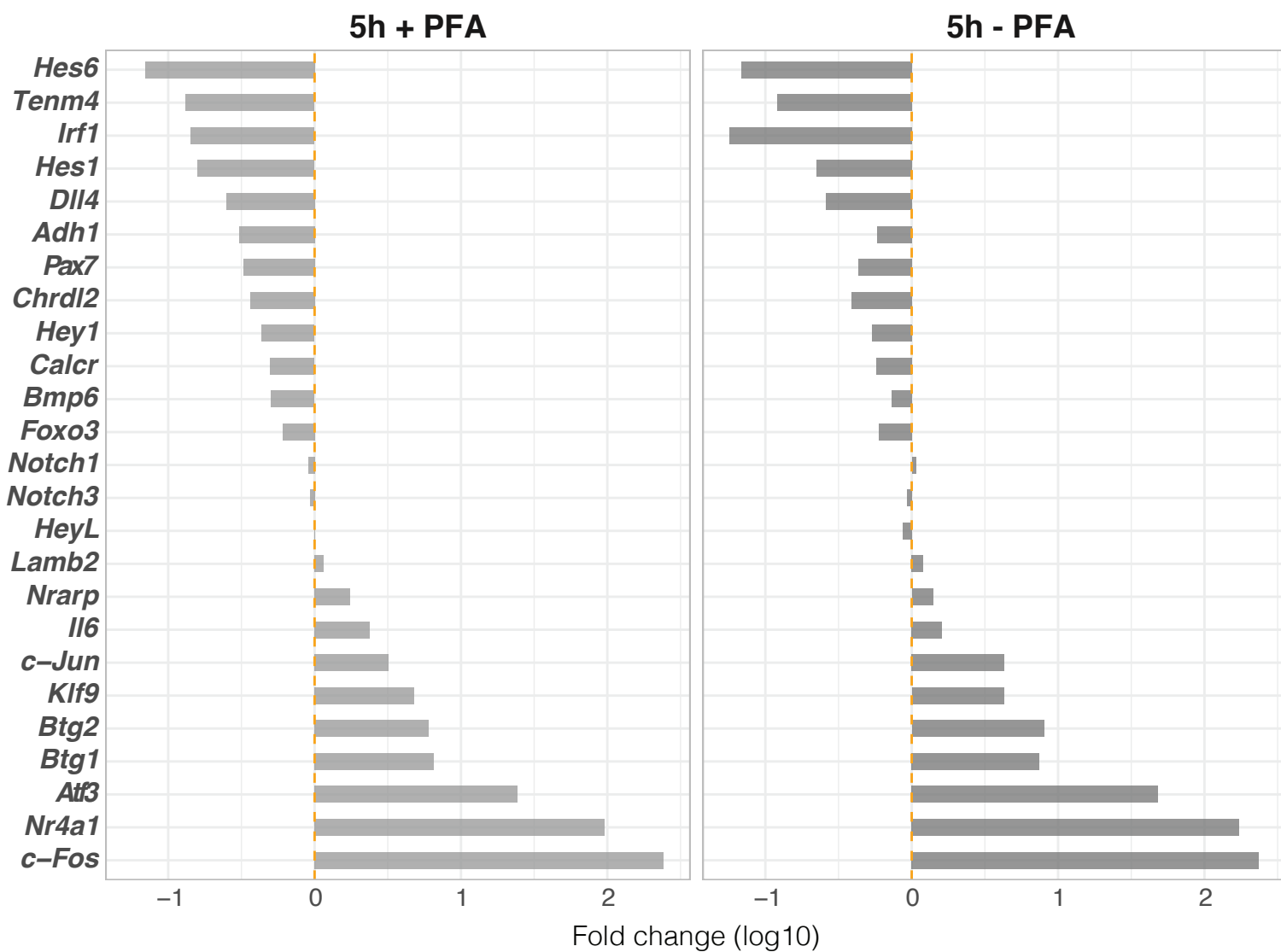

Supplement: Supplementary file 2 — Figure S4. Effect of PFA treatment at different time points in the experimental procedure. Top: Schematic showing the simplified experimental procedure of cell fixation before or after muscle dissociation/cell sorting. “0 h” refers to cells fixed prior to muscle dissociation and cell sorting, while “5 h” refers to cells that undergo muscle dissociation and cell sorting prior any treatment (+/− PFA). Bottom: Barplots showing the effect of PFA treatment after muscle dissociation/cell sorting. Bars represent the fold change (in Log10) of expression between freshly isolated QSCs (0 h + PFA) and QSCs after muscle dissociation/cell sorting with or without PFA treatment, 5 h + PFA and 5 h –PFA, respectively. (PDF 445 kb) [file 13395_2018_165_MOESM4_ESM.pdf]
